# The Factor of Australia in British Foreign Policy

**DOI:** 10.1134/S1019331622100070

**Published:** 2022-09-22

**Authors:** K. A. Godovanyuk

**Affiliations:** Institute of Europe (IE), Russian Academy of Sciences, Moscow, Russia

**Keywords:** Global Britain, Australia, United States, China, Indo-Pacific, defense cooperation, naval drills, AUKUS, Five Power Defense Arrangements, cybersecurity

## Abstract

The Australian component of UK foreign policy in the context of the changing world order is outlined. It is highlighted that, in a value and ideological sense and due to the common Anglo-Saxon identity, London assigns Canberra a key role in the coalition of like-minded countries (“network of liberty”); in geostrategic terms, it perceives Australia as a platform to expand the UK influence in the Indo-Pacific. At present, the “special” partnership between the two countries is underpinned by a number of new agreements, including a “historical” trade deal aimed at strengthening economic ties and in-depth political, diplomatic, and defense cooperation, based on a new military alliance, AUKUS. At the same time, the traditional pragmatism inherent in the foreign policy of Australia, which positions itself as a reliable international actor, is being replaced by increasing military–political and economic dependence, which plays into the hands of London. Coming closer with Australia also allows Britain to present itself as the key extraregional player in the system of anti-Chinese alliances in the Indo-Pacific, with Washington and Canberra in the forefront.

## INTRODUCTION

After Brexit, London is actively restoring alliances that have weakened for various reasons and is counting on rapprochement with traditional partners through political, defense, and economic agreements, as well as value systems aimed at preserving the liberal world order. This implies strengthening bilateral dialogue with like-minded countries, “connecting” allies to the international agenda that meets British foreign policy interests, and concluding new trade agreements [*Britain after Brexit*, 2021, pp. 13−34; Portanskii, 2020], as well as military−political partnerships in the Euro-Atlantic and Indo-Pacific in accordance with the new national security strategy [Anan’eva and Godovanyuk, 2021].

London builds situational alliances with individual countries (Turkey) and considers the states of Northern and Eastern Europe primarily as allies in the geopolitical confrontation with Russia; as for India and Brazil, it evaluates ties with them proceeding from its long-term geostrategic and geoeconomic interests. Australia stands out in the modern system of Britain’s alliances; partnership with it is determined by a complex of historical, economic, political, military, and strategic interests, based on a common identity and assessment of threats to the modern world order.

## PREREQUISITES FOR PARTNERSHIP: COMMON IDENTITY AND FOREIGN POLICY TRADITIONS

In intellectual discussions, Brexit has brought to the fore questions of identity, reflecting myths about the confrontation between the “Anglo-Saxon” and “European” nature of British foreign policy [Vucetic, 2022].

The debate continues on whether special attention to the “Anglosphere”^1^ was an inevitable consequence of leaving the European Union or its cause, as the Brexiters claimed. The opinions of experts concerning what should be viewed as the Anglosphere vary. It was perceived either as a reference to Britain’s imperial past [Hill, 2019] or as a synonym for the Five Eyes (United States, Britain, Australia, Canada, and New Zealand) intelligence alliance. Some foreign studies view the community of English-speaking countries as a conditional geopolitical actor, the weight of which in world politics has increased owing to Brexit [Peters, 2021]. This mindset began to dominate in the strategic planning of the United Kingdom against the backdrop of the discourse about distancing itself from the European Union and strengthening Atlanticism and its interpretation in the Eastern Hemisphere—promoting the American interpretation of security in the Indo-Pacific.

As opposed to the former metropole, Australia has no complex about a special role on the world stage, perceiving the Pacific Ocean and Southeast Asia as the sphere of its priorities. In 1988, Australian Foreign Minister G. Evans (Labor Party) put forward the concept of “good international citizenship” as the basic setting of foreign policy [Evans, 2022]. While modernizing their political course, the British “New Labor” adapted the Australian concept to the tasks of the “Third Way,” defining “an ethical foreign policy” as their goal [Wheeler and Dunne, 1998]. Later, the ideological framework of the British strategy underwent several transformations, acquiring the features of “Global Britain” as a symbol of the rejection of a Eurocentric foreign policy. Australian foreign policy was not characterized by ideological “swings,” and its adaptation to the changing international environment was more inert. Foreign and Russian authors are unanimous in their assessments that in international relations Canberra willingly acts as a “middle power”^2^ [Ungerer*,* 2007; Aleshin, 2020].

Australia has never perceived the association of the former British colonies and dominions as the main line of foreign policy, although it is the Commonwealth of Nations that remains a symbol of the inseparable history and common identity, and hence the common interests of London and Canberra. Researchers noted that Australia’s actions in the logic of a “good international citizen” were clearly manifested precisely within the Commonwealth, where it even managed to act as a mediator [Bridge, 2006].

Over the past 20 years, the country has positioned itself as part of the collective West, participating in NATO military missions—for example, in Afghanistan in 2001. In June 2012, Australia signed a Political Declaration with the North Atlantic Alliance, which was followed by special programs of individual partnership and cooperation in 2013 and 2017 (Individual Partnership and Cooperation Programs). In 2014, Canberra received the status of a NATO Enhanced Opportunities Partner. It subsequently became part of the global coalition against ISIS/Daesh,^3^ acted as an operational partner of the alliance in Iraq, and contributed to NATO antipiracy missions.

In 2014, the former British dominion adopted anti-Russian sanctions in connection with the reunification of Crimea with the Russian Federation, and in 2018, amid the Skripal’ scandal, it expelled two Russian diplomats. Australia, as part of the Anglo-Saxon world, shares the principles of the neoliberal world order with its center in Washington against the backdrop of the deepening split between the “collective West” and the countries that challenge it (Russia, China, etc.).

Britain, which positions itself as a “force for good” and defender of democratic values, has taken the initiative to accept Australia into the “club of Western democracies” (D10). In response, Australia’s Prime Minister S. Morrison proposed on the eve of the G7 summit in 2021^4^ to restore the harmony of the liberal world order, of which Canberra sees itself as a part.^5^ In connection with the special military operation of the Russian Federation on the territory of Ukraine, Australia adopted several packages of anti-Russian sanctions against banks; legal entities; and individuals, including journalists.

With the advent of the concept of a secure Indo-Pacific, Canberra’s claim to regional leadership became more obvious, which can be explained, among other things, by its geographical position at the intersection of the Indian and Pacific oceans (at the center of the geopolitical construct of the Indo-Pacific Region (IPR)). In 2013, Australia was the first to include the term *Indo-Pacific* in its defense strategy.

Although Australia has practically formed a Sinophobia-based internal political consensus [Brophy, 2021], researchers are increasingly expressing doubts that Canberra will be able to maintain its role as a “good international citizen” in the context of growing confrontation with China [Abbondanza, 2021]. In 2018, Australia was one of the first countries of the collective West to ban officially the use of telecommunications equipment from Huawei and ZTE. Relations with the Celestial Empire deteriorated further after the Australian government called in April 2020 for an international comprehensive investigation into the causes of the COVID-19 pandemic and China’s role in it.^6^ Under such conditions, the support of nonregional allies that claim leadership in the region has become vital for Australian foreign policy.

At the same time, upon revising its foreign policy priorities as a consequence of Brexit, Britain has also identified the Indo-Pacific as a zone of its own interests [Godovanyuk, 2020]. In the Integrated Review on national security (March 2021), London recognized the special role of middle powers in the transforming world order, and Australia as a critical ally in the region.

## THE BRITISH−AUSTRALIAN PARTNERSHIP: THE ECONOMIC ASPECT

Australia was the first of the countries of the collective West to take the blow of Beijing’s response to the sanctions, demonstrating in practice its “decoupling” from the Celestial Empire. In 2020, trade with China accounted for 29% of all trade and 39% of Australian exports.^7^ However, in response to the open anti-Chinese conduct, China reduced the volume of imports from Australia by imposing duties on a number of goods (barley, wine, beef, cotton, and iron ore). The scale of China’s boycott of Australian goods was unprecedented, affecting 13 sectors. The most painful were the restrictive measures against the exports of coal, almost a quarter of the volume of international sales of which came from China.^8^

In an effort to minimize the negative effect on its economy, Canberra in 2021 resumed negotiations on a trade agreement with the EU, which had been stalling since 2018. The trade agreement with London, the parameters of which have been worked out by a special working group since 2016, has come to the fore. Britain is the second largest foreign investor in the Australian economy after the United States. However, trade volumes remain very modest. In 2020, Australia became London’s seventh trading partner outside the EU with a turnover of £13.9 billion (exports amounted to £9.8 billion, and imports, to £4.1 billion). For comparison, the annual volume of trade of the United Kingdom with the EU is £660 billion. The general parameters of the Free Trade Agreement between London and Canberra were agreed in June 2021, and in December 2021 the document was signed.^9^ However, the agreement will enter into force only after discussion in parliament, which, according to various estimates, may continue until 2023.

For Britain, the deal was the first “from scratch” after the end of the transition period in relations with the EU. All previously concluded trade agreements (with Japan, Canada, etc.) were “carbon copies” of Brussels’ agreements with third countries, which previously had included London. The British government declared the trade deal with Canberra a priority, guided by interests that are not limited to economic reasons. Politically, the agreement is intended to demonstrate the success of the independent trade policy of “Global Britain.” Emphasizing the special character of the deal, the British establishment confirmed the exclusive status of Australia in their own foreign policy priorities. In June 2021, Minister of International Trade L. Truss, in particular, called the agreements historic and bound to become the “gold standard” of post-Brexit trade agreements. However, according to analysts, the maximum possible economic benefit for Britain will be from 0.01 to 0.02% of GDP (£500 million over 15 years under an optimistic scenario).^10^

Negotiations were greatly facilitated by the fact that Australia is a relatively small and open economy, which has established minimal trade barriers to goods and investment from the United Kingdom. The parties agreed to remove all tariffs on exported British goods, particularly on cars, whiskey, textile products, etc. (turnover for about £4 billion), and on almost all Australian goods exported to Britain.

The deal opens the British livestock and agriculture market (beef, mutton, dairy products, sugar, etc.) for Australia, which is still subject to quotas. It was decided that after the agreement has entered into force, tariffs and quotas, for example, for beef and mutton, will be reduced within 15 years and for sugar, within 8 years. However, the agreement faced opposition on the part of British farmers,^11^ for whom the deal was an “unpleasant Christmas present.” They expressed fears that market liberalization would hit British manufacturers, increase imports by several times, and intensify separatist sentiments in Scotland. In addition, the deal with Australia, as well as the hypothetical trade agreement with the United States, raised concerns about the possible lowering of production standards (Australian farmers use pesticides) since the document does not spell out a standard control mechanism.

The developers hope that the trade agreement will stimulate online commerce and create opportunities for the free movement of digital data and a common “electronic environment” in the field of commercial services. The latter item is of concern to the British due to fears that British standards for regulating the Internet environment may be lowered.

The agreement does not provide for the freedom of the movement of labor between Britain and Australia, as was expected, but provides for measures that greatly facilitate the movement of young people (from 18 to 35 years old). Under a special mobility scheme, Australians will be able to stay in Britain for three years.

More importantly, the trade deal, along with the previous one with Japan, paves the way for the United Kingdom to enter the Comprehensive and Progressive Trans-Pacific Partnership (CPTPP), negotiations on which began in June 2021.^12^ UK trade volumes with countries of this group in 2019 amounted to £111 trillion, an increase of 8% since 2016. London expects Canberra to provide lobbying support for its entry into this economic bloc.

## MILITARY−POLITICAL COOPERATION: THE REGIONAL ASPECT

Since the second half of the 20th century, Britain, by decision of the Labor government of H. Wilson, withdrew most of the military contingent from the region “east of Suez,” losing its strategic positions in the Indian and Pacific oceans. In 1966, Australian Prime Minister H. Holt called such a decision a historical error since the “strength, stability, and peaceful progress” of the region needed the “moral, material, and even military help of the United Kingdom.”^13^ Experts pointed out that it was the reliance on Australia that allowed London to feel the terra firma in the region [Howard, 1966]. The reorientation of the former dominions towards cooperation with Washington, reinforced in 1951 by the military−political agreement of Australia, New Zealand, and the United States (ANZUS), in fact meant the “narrowing” of the zone of influence of the United Kingdom to the European continent.

However, Britain did not leave the region fully. Thus, according to experts, the Five Power Defense Agreement (Australia, Malaysia, New Zealand, Singapore, and the United Kingdom), signed in 1971, allowed Britain to join the “ecosystem” of defense alliances in Southeast Asia. The agreements differ from NATO’s principle of collective defense: they oblige countries to consult in the event of an attack on one of them. Today, such formats, along with the Five Eyes Alliance, are of particular value for British foreign policy, although, as many point out, they remain the legacy of the empire and the Cold War era.

An increased interest in military−political cooperation with Australia was demonstrated by the coalition government of conservatives and liberal democrats (2010−2015), which, long before Brexit, had pursued a policy of diversifying trade and political partners. In 2013, the countries signed an agreement on defense and military cooperation (Australia–United Kingdom Defense and Security Cooperation Treaty).^14^ After a long break, the AUKMIN ministerial dialogue was revived^15^ between the heads of the foreign policy and defense departments.

In the strategic documents of Australia, the priority nature of cooperation with Britain is fixed. Thus, the 2016 Defense White Paper notes that British−Australian relations are based on historical and cultural ties. Both countries are for a “rules-based order,” coordinate approaches to ensuring international security, and agree on threat assessments.^16^ The 2017 Australian Foreign Policy White Paper names Britain as the most important international partner.^17^

Since 2017, at the suggestion of Washington, Canberra has been actively involved in anti-Chinese political formats in the region. For example, it participates in a four-sided security dialogue with India, the United States, and Japan, reanimated by D. Trump. With the advent of the Biden administration, the role of military−political alliances in the Pacific increased in connection with the American vision of the concept of a “free and open Indo-Pacific” [Wallis and Powles, 2021]. In line with the general trend towards the militarization of the region, the updated Australian Defense Strategy of 2020 provides for a significant increase in military spending (over $270 billion over the next ten years).^18^ This trend opens additional “windows of opportunity” to strengthen the strategic position of Global Britain as a “natural ally” of Canberra in the long term and brings direct benefits to the British military−industrial complex.

Since 2017, the parties have been holding a high-level ministerial dialogue on defense and defense cooperation, the Defense Industry and Capability Dialogue, aimed at close cooperation in the defense industry. A year later, the British BAE System won a tender for the development and construction of nine new-generation Hunter-class frigates based on the British Type 26 Global Warship for the Royal Australian Navy. Production will be handled by ASC Shipbuilding in Osborne, South Australia (the birthplace of Australian shipbuilding). The most important aspects of cooperation were noted in a special memorandum of the defense ministers of the two countries in October 2020.^19^

The return of Britain to the region was expressed primarily in naval activity with the support of the closest allies. The most important event on the 2021 agenda was the naval maneuvers of the aircraft carrier group, announced back in 2017 by Foreign Minister B. Johnson during his visit to Sydney. Britain actively coordinated the maneuvers in the IPR with Australia. Moreover, it was decided that two British warships, HMS *Spey* and HMS *Tamar*, would be permanently stationed in the region, supported by the Australian Navy.

In addition, large-scale military drills of Australia and the United States began in July 2021, which were also joined by 11 states, including the United Kingdom.^20^ A month later, the navies of the two countries actively participated in the Bersama Gold exercise to mark the 50th anniversary of the Five Power Defense Agreement.

The culmination of the international agendas of the two countries was the announcement of the creation of a trilateral security pact between Australia, the United States, and Britain in the fall of 2021, which will legitimize Britain’s presence in the IPR in the long term. The agreements involve in-depth cooperation in the field of the exchange of military developments and technologies, as well as the coordination of defense and diplomatic cooperation in the Indo-Pacific. The first step of this partnership was the decision to assist Canberra in the construction of eight nuclear submarines, in connection with which, in November 2021, the parties signed an agreement on the transfer of relevant nuclear technologies.^21^ In April 2022, it was announced that the next stage of trilateral cooperation would be joint work on hypersonic weapons.^22^ The agreement on AUKUS is aimed at ensuring that the closest allies continue to involve Australia in the arms race in the region and escalate tensions, which the Chinese authorities have repeatedly called a manifestation of “cold war thinking.” Chinese media have described Australia as a “gangster” that promotes an “axis of white supremacy” in “a US-centered mafia-styled community.”^23^

AUKUS with broader goals of military and defense cooperation is a triangle of privileged partners, autonomous from the Five Eyes Alliance, united by common goals in the region. The constant solidarity with the American interpretation of security in the IPR [Scott, 2013], according to experts, is changing the foreign policy tradition of Australia, which upsets the balance between the behavior of a “middle power” and a “dependent ally” [Taylor, 2020]. In fact, Australia is gradually turning from a “good international citizen” into a player whose behavior is subordinated to Washington’s strategy. London, in turn, acts as a force that cements the union of the three powers; seeks to try on the role of a bridge, tested in the transatlantic direction; and, by appealing to a common identity, tries to present itself as a party capable of influencing Canberra. Military experts do not rule out that, to demonstrate its presence, Britain will deploy a military contingent in the former dominion, where the US military presence has already increased since 2018 (in particular, in the capital of the Northern Territory, the city of Darwin).

Further strengthening of the bilateral dialogue was confirmed by the next round of negotiations in the AUKMIN format in January 2022.^24^ The visit of two key members of the British Cabinet (Foreign Minister Truss and Defense Minister B. Wallace) to Australia took place against the backdrop of an aggravation of the military−political situation in Europe and the circulating reports of an allegedly planned “Russian invasion of Ukraine.” Symbolic was Truss’ speech at the leading Australian think tank, the Lowy Institute, where she called Australia the closest ally “in defense of freedom and democracy around the world.”^25^ This speech echoed the keynote speech of Truss at Chatham House in December 2021, where she called the “network of liberty,” which will be created with the closest allies, a priority for London.^26^

The results of the British−Australian AUKMIN negotiations were of applied character as well. In particular, the parties agreed on the Strategic Infrastructure and Development Dialogue. The new agreement provides support for high-tech projects, such as disaster- and climate-resilient infrastructure in the IPR. The countries get access to one another’s training grounds and research programs and are engaged in joint military developments on an ongoing basis.

Finally, still another future area of cooperation is the Cyber and Critical Technology Partnership. In February 2022, during the virtual summit of the two prime ministers, an agreement was signed to strengthen the contribution of Britain to cybersecurity, maritime security, and countering threats from states, in the amount of £25 million.^27^ The focus of the parties is on global technology supply chains and countering malicious activity in cyberspace and countering “malicious state actors” in line with London’s efforts to create a “network of liberty,” including in the information sphere.

The parties are actively expanding cooperation in the field of science and technology: the British government will support the Australian fintech company PEXA, and the British energy company Octopus will work on renewable energy projects in Australia.

In general, London positions itself as the preferred European partner in the IPR, competing with Beijing and emphasizing common values, historical destiny, and identity with Canberra.

## CONCLUSIONS

The complex of historical, political, economic, and geostrategic factors, aimed at overcoming intense competition in modern international relations, has led to increased attention in London to the former dominion. As conceived by the developers of the Global Britain concept, the emphasis on the defense component of the partnership with Canberra should stress the exceptional role of London in ensuring security in the megaspace from the Euro-Atlantic to the Indo-Pacific.

Britain seeks to influence Australia using the rhetoric of a “special” and “natural” partnership. Johnson’s cabinet promoted the so-called Australian-style trade deals and Australian-style immigration reform, which was most often just a figure of speech designed to emphasize the special importance of Canberra in British politics and the similarity of their interests. Since the EU membership referendum, the British establishment has become more vocal about the similarities between the British and Australian government models and, more broadly, about adherence to the neoliberal system of international relations, to which the Chinese media reacted with a rhetorical question whether Britain would be the next target of large-scale Chinese sanctions.^28^ The paradox of London’s policy for a long time was that the country did not formulate an unambiguous line in relation to the Celestial Empire. For example, the United Kingdom was the last among the Five Eyes allies to take a hard line on Chinese tech giant Huawei, which shows the forced nature of that decision.

At the same time, the British leadership has repeatedly stated that London will support Canberra in its confrontation with China. Washington and London are counting on Australia as a “stronghold” in the Indo-Pacific, which was formally confirmed by the AUKUS military−political alliance, which cements the common course of the allies to contain Beijing in an attempt to shift the balance of power in their own favor in the region.

The Australian government’s course of confrontation with China is drawing criticism from the opposition since the country risks losing international economic competition under the Chinese sanctions, as well as being involved in a direct conflict with Beijing. The role of the United Kingdom, despite attempts to mediate in the Indo-Pacific and be Canberra’s “special partner,” remains subsidiary to American policy, while London’s willingness to engage in a direct military clash in the region is unlikely.

## Footnotes

1 The *Anglosphere* is a collective term for English-speaking countries (United States, Britain, Canada, New Zealand, and Australia) [Wellings and Mycock, 2019].

2 International relations theorists began to use the term *middle powers* after World War II, primarily to refer to the status of Australia and Canada. In the absence of a strict definition, various schools of international relations offer their own interpretations of this term. In the ideological paradigm of neorealism, which dominated until the mid-20th century, the dichotomy of *great powers* vs. *others* was generally accepted. Later, *middle powers* were mainly classified according to functional, behavioral, and hierarchical characteristics [Chapnick, 1999, 73−82].

3 A terrorist organization banned in the Russian Federation.

4 In 2021, Australia, along with India and South Africa, was invited to Cornwall for the G7 summit. According to media reports, Morrison expressed disappointment that he had no chance to meet with US President J. Biden on a bilateral basis since British Prime Minister B. Johnson joined their conversation.

5 “We Aussies want to use our G7 invite to help build an enduring liberal order,” Telegraph, June 11 (2021). https://www.telegraph.co.uk/news/2021/06/11/aussies-want-use-g7-invite-help-build-enduring-liberal-order/.

6 “Marise Payne calls for global inquiry into China’s handling of the coronavirus outbreak,” ABC News, Apr 19 (2020). https://www.abc.net.au/news/2020-04-19/payne-calls-for-inquiry-china-handling-of-coronavirus-covid-19/12162968.

7 Australia’s trade in goods with China in 2020. Australian Bureau of Statistics. https://www.abs.gov.au/articles/australias-trade-goods-china-2020.

8 Australia annually produces about 500 million tons of coal, most of which (up to 75%) it exports. In 2019, the country exported $64 billion worth of coal, which provided up to 8.3% of GDP.

9 UK−Australia Free Trade Agreement. https://www.gov.uk/government/collections/uk-australia-free-trade-agreement.

10 UK−Australia free trade agreement: the UK’s strategic approach. https://www.gov.uk/government/publications/uks-approach-to-negotiating-a-free-trade-agreement-with-australia/uk-australia-free-trade-agreement-the-uks-strategic-approach.

11 “Farmers’ opposition to UK−Australia trade deal grows,” BBC, June 2 (2021). https://www.bbc.com/news/science-environment-57268681.

12 Britain launches negotiations with £9 trillion Pacific free trade area, June 21 (2021). https://www.gov.uk/government/news/ britain-launches-negotiations-with-9-trillion-pacific-free-trade-area.

13 Will AUKUS cement ‘Global Britain’? ASPI Strategist. https://www.aspistrategist.org.au/will-aukus-cement-global-britain.

14 “Australia–UK defence arrangements,” ASPI Strategist. https://www.aspistrategist.org.au/australia-uk-defence-arrangements.

15 Britain and Australia began regular AUKMIN consultations in 2006.

16 Defence White Paper, 2016. https://www.defence.gov.au/whitepaper/Docs/2016-Defence-White-Paper.pdf, pp. 137−138.

17 2017 Foreign Policy White Paper. https://www.dfat.gov.au/publications/minisite/2017-foreign-policy-white-paper/fpwhitepaper/foreign-policy-white-paper/chapter-six-global-cooperation/ our-global-partnerships.html.

18 Defending Australia and its interests, Prime Minister of Australia. https://www.pm.gov.au/media/defending-australia-and-its-interests.

19 Australia and the United Kingdom cooperate on frigate programs, Australian Government, Ministry of Defence. https://www.minister.defence.gov.au/minister/lreynolds/media-releases/australia-and-united-kingdom-cooperate-frigate-programs.

20 “11 nations participate in massive US−Australia military drill,” Times of India, July 16 (2021). https://timesofindia.indiatimes.com/world/rest-of-world/11-nations-participate-in-massive-us-australia-military-drills-as-tensions-escalate-in-indo-pacific/articleshow/84458710.cms.

21 UK/Australia/USA: Agreement for the Exchange of Naval Nuclear Propulsion Information. https://www.gov.uk/government/publications/ukaustraliausa-agreement-for-the-exchange-of-naval-nuclear-propulsion-information-ms-no82021.

22 AUKUS Leaders’ Level Statement: April 5, 2022. https://www.gov.uk/government/news/aukus-leaders-level-statement-5-april-2022.

23 Chinese state media calls Australia a “gangster” as trade war flares, February 26, 2021. https://www.news.com.au/world/five-eyes-china-accuses-australia-of-being-part-of-an-axis-of-white-supremacy/news-story/14599f6ca8e6100632b30557c2105a75.

24 Australia−UK ministerial meeting, January 21, 2022. https://www.gov.uk/government/publications/aukmin-2022-joint-statement/australia-uk-ministerial-meeting-joint-statement.

25 Foreign Secretary Liz Truss’ speech to the Lowy Institute. https://www.gov.uk/government/speeches/foreign-secretarys-speech-to-the-lowy-institute.

26 Foreign Secretary Liz Truss and the UK’s foreign policy priorities, Chatham House, December 8, 2021. https://www.chathamhouse.org/events/all/members-event/foreign-secretary-liz-truss-and-uks-foreign-policy-priorities.

27 Joint statement on UK−Australia virtual summit: February 16, 2022. https://www.gov.uk/government/news/joint-statement-on-uk-australia-virtual-summit-16-february-2022.

28 “Will UK be the next Australia and inflict damage on itself?,” Global Times, Feb. 18 (2021). https://www.globaltimes.cn/ page/202102/1215833.shtml.
